# Gut microbiota-derived butyrate improved acute leptospirosis in hamster *via* promoting macrophage ROS mediated by HDAC3 inhibition

**DOI:** 10.1128/mbio.01906-24

**Published:** 2024-09-17

**Authors:** Xi Chen, Xufeng Xie, Ni Sun, Xin Liu, Jiuxi Liu, Wenlong Zhang, Yongguo Cao

**Affiliations:** 1 Department of Clinical Veterinary Medicine, College of Veterinary Medicine, Jilin University, Changchun, China; 2 State Key Laboratory for Diagnosis and Treatment of Severe Zoonotic Infectious Diseases, Key Laboratory for Zoonosis Research of the Ministry of Education, Institute of Zoonosis, and College of Veterinary Medicine, Jilin University, Changchun, China; Cornell University College of Veterinary Medicine, Ithaca, New York, USA

**Keywords:** butyrate, leptospirosis, macrophage, HDAC3, ROS

## Abstract

Leptospirosis is a re-emerging worldwide zoonotic disease. Infected patients and animals often exhibit intestinal symptoms. Mounting evidence suggests that host immune responses to bacterial infection are closely associated with intestinal homeostasis. Our previous research has shown that the gut microbiota can protect the host from acute leptospirosis, while the specific bacterial metabolic mediators participating in the pathogenesis remain to be identified. Short-chain fatty acids (SCFAs) are metabolites produced mainly by the gut microbiota that play a role in immune regulation. However, whether SCFAs are the key to protecting the host against leptospirosis and the underlying regulatory mechanisms are unknown. In this study, our results showed that the SCFA butyrate is involved in ameliorating leptospirosis. The depletion of SCFAs by antibiotic cocktail treatment reduced survival time after *Leptospira* infection while supplementation with butyrate but not acetate or propionate significantly amelioration of leptospirosis. *In vitro* experiments showed that butyrate treatment enhanced the intracellular bactericidal activity mediated by reactive oxygen species (ROS) production. Mechanistically, butyrate functions as a histone deacetylase 3 inhibitor (HDAC3i) to promote ROS production *via* monocarboxylate transporter (MCT). The protection of butyrate against acute leptospirosis mediated by ROS was also proven *in vivo*. Collectively, our data provide evidence that the butyrate-MCT-HDAC3i-ROS signaling axis is a potential therapeutic target for acute leptospirosis. Our work not only interprets the microbial metabolite signaling involved in transkingdom interactions between the host and gut microbiota but also provides a possible target for developing a prevention strategy for acute leptospirosis.

**IMPORTANCE:**

Leptospirosis is a worldwide zoonotic disease caused by *Leptospira*. An estimated 1 million people are infected with leptospirosis each year. Studies have shown that healthy gut microbiota can protect the host against leptospirosis but the mechanism is not clear. This work elucidated the mechanism of gut microbiota protecting the host against acute leptospirosis. Here, we find that butyrate, a metabolite of gut microbiota, can improve the survival rate of hamsters with leptospirosis by promoting the bactericidal activity of macrophages. Mechanistically, butyrate upregulates reactive oxygen species (ROS) levels after macrophage infection with *Leptospira* by inhibiting HDAC3. This work confirms the therapeutic potential of butyrate in preventing acute leptospirosis and provides evidence for the benefits of the macrophage-HDAC3i-ROS axis.

## INTRODUCTION

Leptospirosis is a re-emerging zoonosis caused by the pathogenic *Leptospira* spp. *Leptospira* infects humans and animals worldwide, causing approximately 1 million human infections and 60,000 deaths every year (1, 2). The clinical manifestations of leptospirosis in humans, along with those in other susceptible mammals range from mild flu-like symptoms such as fever and headache to severe pulmonary hemorrhage, multiorgan failure, and even death (3).

For a long time, intestinal bleeding has been a common but neglected symptom in infected patients and animals (4–6). Recently, Inamasu et al. demonstrated that *Leptospira* could colonize the intestinal tract in hamsters and cause submucosal hemorrhage (in an animal model of acute leptospirosis) (6). Our previous research has shown that the gut microbiota could protect the host from acute leptospirosis, while the specific bacterial metabolic mediators participating in the pathogenesis remain to be identified (7, 8).

A healthy gut environment, shaped by the presence of a healthy functional microbial ecosystem, is fundamental for directing the immune system toward homeostasis (9). It is reported that 95% of the diseases are related to the dysbiosis of the gut microbiota, such as gastrointestinal diseases, metabolic diseases, and infectious diseases. Our previous studies have found that gut microbiota is closely related to leptospirosis, and gut microbiota protects the host against leptospirosis by regulating the antibacterial function of host macrophages but the detailed mechanism is still unclear. So how does the gut microbiota regulate the host immune response and then affect the host systemic *Leptospira* infection? Studies have found that gut microbiota closely interacts with the host mainly through small molecule metabolites, affecting the occurrence and development of diseases, such as SCFAs. SCFAs as a major class of bacterial metabolites are important regulators of host defense against infections (10). For example, microbiota-derived acetate enhances the host resistance to influenza A virus infection through the gut-lung axis (11). *Bacteroides* spp. mediate colonization resistance to *Salmonella* infection through the production of propionate (12). Butyrate treatment promoted antibacterial activity in intestinal macrophages and restricted bacterial translocation in mice (13). However, whether SCFAs are the key to protect the host against leptospirosis and the underlying regulatory mechanisms are unknown.

In this study, we aimed to explore the role and mechanism of SCFAs in acute leptospirosis. SCFA depletion aggravated leptospirosis while supplementation with butyrate significantly improved *Leptospira* infection, promoting ROS production by inhibiting histone deacetylase (HDAC) 3 mediated by monocarboxylate transporter (MCT). Taken together, our results provide new insights into the relationship between the gut microbiota and leptospirosis and suggest a role for butyrate in developing appropriate therapeutic approaches.

## MATERIALS AND METHODS

### Bacterial strains and animals

The pathogenic *Leptospira interrogans* serovar Lai strain Lai (56601) was grown in liquid Ellinghausen–McCullough–Johnson–Harris (EMJH) medium at 29 ℃, and pathogenicity was maintained by passage in hamsters (14). *Leptospira* underwent less than three passages *in vitro* to guarantee the stability of the pathogenicity of *Leptospira* (15). The number of bacterial cells was determined using a Petroff-Hausser counting chamber and dark-field microscopy (16). Hamsters were provided by the Liaoning Chang Sheng Biotechnology Co. LTD.

### Experimental infection in hamsters

To explore the role of SCFAs in *Leptospira* infection, the gut microbiota was depleted with broad-spectrum antibiotics (Abx) as previously described (17). Six-week-old female hamsters were intragastrically gavaged with Abx [1 g/L ampicillin, 1 g/L metronidazole, 1 g/L neomycin sulfate (purchased from Sigma), and 0.5 g/L vancomycin (BiochemPartner, China)] for 7 days. Then, feces were aseptically collected for further examination and the hamsters were infected with 10^6^ leptospires.

For analysis of the role of SCFAs in *Leptospira* infection, the 6-week-old female hamsters received acetate, propionate, and butyrate (150 mM final concentration) in their drinking water for 21 days. Hamsters aged 6 weeks were inoculated with 10^6^ leptospires by intraperitoneal injection. After infection with leptospires, all hamsters were observed no less than three times per day for a period of 21 days, during which time seriously sick hamsters appeared moribund and were then humanely euthanized by CO_2_. Six hamsters from each group were humanely euthanized 6 days after infection, and their blood, livers, lungs, and colons were aseptically collected and kept at −80°C. Surviving hamsters were humanely euthanized after 21 days using CO_2_.

For analysis of the role of ROS in the protection of butyrate against acute leptospirosis, the hamsters received butyrate in their drinking water for 21 days. Whereafter, the hamster was treated with NAC during the last 7 days of the 21 days. The survival rate of hamsters after infection with *Leptospira* was recorded by the method described above. Six hamsters from each group were humanely euthanized 6 days after infection, and their colons were aseptically collected and kept at −80°C. To analyze the effect of butyrate on the virulence of *Leptospira*, butyrate (1 mM) was added to EMJH medium, and PH was adjusted to 7.3–7.4. *Leptospira* was cultured using EMJH (with or without butyrate) medium for 5 days. Hamsters were inoculated with 10^6^ leptospires by intraperitoneal injection. The survival rate of hamsters after infection with *Leptospira* was recorded by the method described above. In addition, 5 × 10^8^ leptospires were collected by centrifugation at 3,500 × *g* for 10 min and kept at −80°C.

### Cell culture and infection

Primary peritoneal macrophages were isolated and cultured as previously described (18). In brief, 6-week-old hamsters were injected with 2 mL of 3% thioglycolate medium. Three days after the injection, peritoneal macrophages were isolated by washing the peritoneal cavity with PBS and enriched by plating the lavage cells on tissue culture plates in RPMI 1640 supplemented with 10% fetal bovine serum and 1% penicillin and streptomycin at 37°C for 2 h. Then, the plate was washed three times with sterile PBS to remove nonadherent cells. Primary peritoneal macrophages were cultured in RPMI 1640 supplemented with 10% fetal bovine serum, 100 U/mL penicillin, and 100 µg/mL streptomycin in a 5% CO_2_ atmosphere at 37°C. Before infection, the cells were washed three times with RPMI 1640 to remove penicillin and streptomycin.

Cell counting kit 8 (CCK8) assay was used to determine the optimal concentration of butyrate to stimulate peritoneal macrophages. Briefly, cells were seeded and cultured at a density of 5 × 10^3^/ well in 100 µL of medium into 96-well microplates. Then, the cells were treated with various concentrations of butyrate (0, 0.1, 0.5, 1, 5, and 10 mM). After treatment for 24 h, 10 µL of CCK-8 reagent was added to each well and then cultured for 2 h. The absorbance was analyzed at 450 nm using a microplate reader.

For the phagocytosis experiment, cells were incubated with leptospires for 1 h. Gentamicin was added to the culture medium for 1 h to ensure that the bacteria were killed. After trypsinization and three washes with RPMI, the cells were centrifuged at 200 × *g* for 5 min, and the pellets were stored at −80°C for DNA extraction.

For the gentamicin protection assay, we cultured peritoneal macrophages with butyrate (1 mM) medium for 24 h in advance and then carried the gentamicin protection assay. Macrophages were infected with leptospires for 1 h and then incubated for 1 h in a medium containing gentamicin (100 µg mL^−1^) to kill the remaining extracellular bacteria. Then, the cells were incubated in a medium containing gentamicin (25 µg mL^−1^) for another 4 h. The cells were then lysed with 1 mL of distilled water and 100 µL of distilled water was inoculated into 2 mL of EMJH medium. After 6–8 days, the number of bacteria was recorded by a Petroff-Hauser chamber.

To explore the role of ROS in butyrate-enhanced macrophage killing of *Leptospira*, macrophages with butyrate (1 mM) at 37°C for 23 h. NAC was added to the culture medium for 1 h. Then the phagocytosis experiment and gentamicin protection assay were performed as described above.

### ROS detection in peritoneal macrophages

For macrophage ROS staining, the cells were stimulated with 1 mM butyrate, acetate, and propionate for 24 h and infected with *Leptospira* for 8 h. Cellular ROS levels were detected *via* the use of ROS Oxidative Stress reagent. After infection with *Leptospira*, macrophages were washed with prewarmed PBS and stained with 5 µM ROS Oxidative Stress for 30  min. Fluorescence was then observed using a fluorescence microscope. All images are representative of three independent experiments.

### MIC determination

The minimum inhibitory concentration (MIC) of butyrate against *L. interrogans* serovar Lai strain Lai 56601 was determined according to a previous method (19). Briefly, leptospires in the exponential phase were deposited at a final concentration of 2 × 10^6^ leptospires/mL in each well of 96-well plates supplemented with 1 mM butyrate. The final volume of each well was 200 µL. The plates were incubated for 3 days at 30°C, after which 20 µL of Alamar Blue (Invitrogen, Thermo Fisher Scientific) was added to each well. Then, the samples were incubated at 30°C for 2 days. Each strain–drug combination was tested in duplicate, and positive (bacteria and no antibiotics added) and negative (no bacteria added) controls were included in each plate. The results were recorded by a microplate reader (MultiSkan FC, Thermo Fisher Scientific).

### Quantitative real-time PCR (qPCR)

DNA was extracted to determine the leptospiral load in the cells. Briefly, specimens (0.09–0.15 g) of tissues were homogenized with PBS (wt/vol, 1/10). The homogenate was centrifuged at 2,000 rpm and 4°C for 5 min. The supernatant was transferred to a new tube. Then, the mixture was centrifuged at 12,000 rpm and 4°C for 5 min. The tissue, blood (100 µL), and cell pellets were extracted using the TIANamp Bacteria DNA Kit (Tiangen, China) according to the manufacturer’s instructions. The primers specific to LipL32 were used to detect leptospires (Table 1). Total RNA was extracted from cells, resuspended bacterial pellets, and tissues to measure gene expression. Total RNA was extracted using TRIzol following the manufacturer’s instructions. The RNA was reverse transcribed into cDNA using random primers from a TransScript One-Step gDNA Removal Kit and cDNA Synthesis SuperMix (TransGen Biotech, China). The primers used in this study are listed in Table 1. The qPCR was performed using an Applied Bioscience 7500 thermocycler and FastStart Universal SYBR Green Master (Roche Applied Science, Germany). PCR conditions were as follows: 50°C for 2 min and, 95°C for 10 min, followed by 45 cycles of amplification at 95°C for 15 s and 60°C for 60 s. The expression of target genes was normalized to that of glyceraldehyde-3-phosphate dehydrogenase (GAPDH) for cells and tissues and to that of 16S rRNA for *Leptospira* using the threshold cycle (2^−ΔΔ*CT*^) method.

**TABLE 1:** Sequence of primers used for qPCR assays

Hamster gene product	Primer	Sequence (5′→3′)	Identifier
GAPDH	Sense	GATGCTGGTGCCGAGTATGT	(20)
	Antisense	GCCACGCCCACATCATTC	
NOX4	Sense	GCCCAGGTTCCAAGCTCAT	This study
	Antisense	TGGTGACAGGTTTGTTGCTCC	
NOX1	Sense	ATGCTTCGCTTTTATCGCTCC	This study
	Antisense	GCTGAAGCCACGCTTTCG	
Cat	Sense	GGCACATTTTGACAGAGAGCG	This study
	Antisense	GACTGTGGAGAATCGAACGGC	
Gsr	Sense	ATGCGTCCCCAAAAAGGTAAT	This study
	Antisense	CTCACATAGGCATCACGCTTCT	
LipL32	Sense	TCGCTGAAATRGGWGTTCGT	(8)
loa22 flaA2 16S rRNA	Antisense Sense Antisense Sense Antisense Sense Antisense	CGCCTGGYTCMCCGATT TTGTTGTGGTGCGGAAGTCG GGTCCCGAACAAGCAGAAGG CGTCAGAGGATTTGATAGAGTG CCAGGAATTGTAGCGGT AGCACGTGTGTTGCCCTAGACATA GTTGCCATCATTCAGTTGGGCACT	(21) (22) (23)

### ROS staining of colon tissue

The frozen colon tissues were cut with a freezing microtome. The fluorescence quencher and ROS dye solution (SIGMA D7008, 1:500) were added to the prepared frozen spleen tissue sections for 5 min. The slides were placed in PBS (PH 7.4) and washed three times by shaking on a decolorization shaker, for 5 min each. After the sections had dried slightly, DAPI dye solution (Servicebio G1012) was added to the rings, and the slices were incubated for 10 min at room temperature in the dark. The slides were placed in PBS (PH 7.4) and washed three times by shaking on a decolorization shaker, for 5 min each. The slices were sealed with an anti-fluorescence quenching tablet. For microscopic examination and photography, the sections were observed, and images were collected under a fluorescence microscope.

### Quantification of SCFAs by GC-MS

Hamster feces were homogenized for 1 min with 500 µL of water and 100 mg of glass beads, and then centrifuged at 4°C for 10 min at 20,000 × *g*. 200 µL supernatant was extracted with 100 µL of 15% phosphoric acid and 20 µL of 375 µg mL^−1^ 4-methylvaleric acid solution as the IS and 280 µL of ether. Subsequently, the samples were centrifuged at 4°C for 10 min at 20,000 × *g* after vortexing for 1 min, after which the supernatant was transferred to a vial prior to gas chromatography-mass spectrometry (GC-MS) analysis. The GC analysis was performed on a trace 1300 gas chromatograph (Thermo Fisher Scientific, USA). Mass spectrometric detection of metabolites was performed on an ISQ 7000 (Thermo Fisher Scientific, USA) with electron impact ionization mode.

### Statistical analysis

The data are expressed as the mean ± standard deviation. One-way analysis of variance was used for statistical analysis, followed by the Newman‒Keuls test. Survival differences between the study groups were compared using the Kaplan–Meier log-rank test. Differences were considered significant at *P* < 0.05.

## RESULTS

### The microbiota metabolite butyrate improved leptospirosis in hamsters

**Fig 1:**
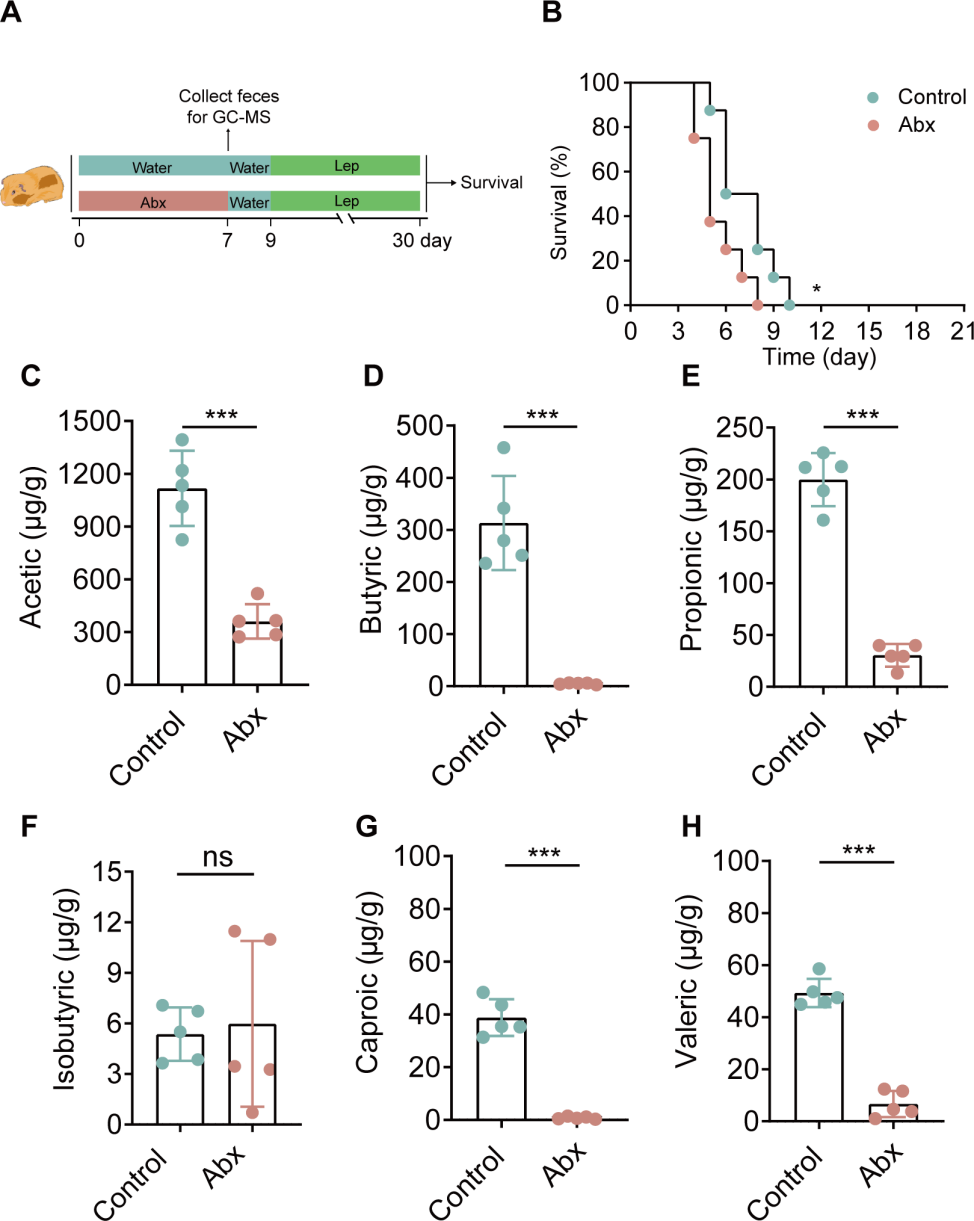
The microbiota metabolite butyrate protected against acute leptospirosis in hamsters. (**A**) Flow diagram of the experiment. Feces were collected from Abx-treated hamsters and control hamsters on the 7th day for metabolomics. (**B**) Survival curves of the Abx group (*n* = 8) and control group (*n* = 8) after infection with 1 × 10^6^ leptospires. (**C**) Fecal acetic (**C**), butyric (**D**), propionic (**E**), isobutyric (**F**), caproic (**G**), and valeric (**H**) levels in hamsters (*n* = 5). The data are shown as the mean ± SEM. Statistical significance was determined using the Wilcoxon rank-sum test. *P < 0.05. ***P < 0.001. ns not significant.

Given that SCFAs are derived from gut microbiota metabolism (24), we sought to deplete the gut microbiota using Abx to reduce the level of SCFAs. (Fig. 1A). The results showed that Abx treatment significantly reduced SCFA levels in the feces (Fig. 1C through H). In addition, compared with those in the control group, gut microbiota depletion accelerated death in hamsters (Fig. 1B). These results suggest that the SCFAs from gut microbiota may help hamsters defend against *Leptospira* infection.

**Fig 2:**
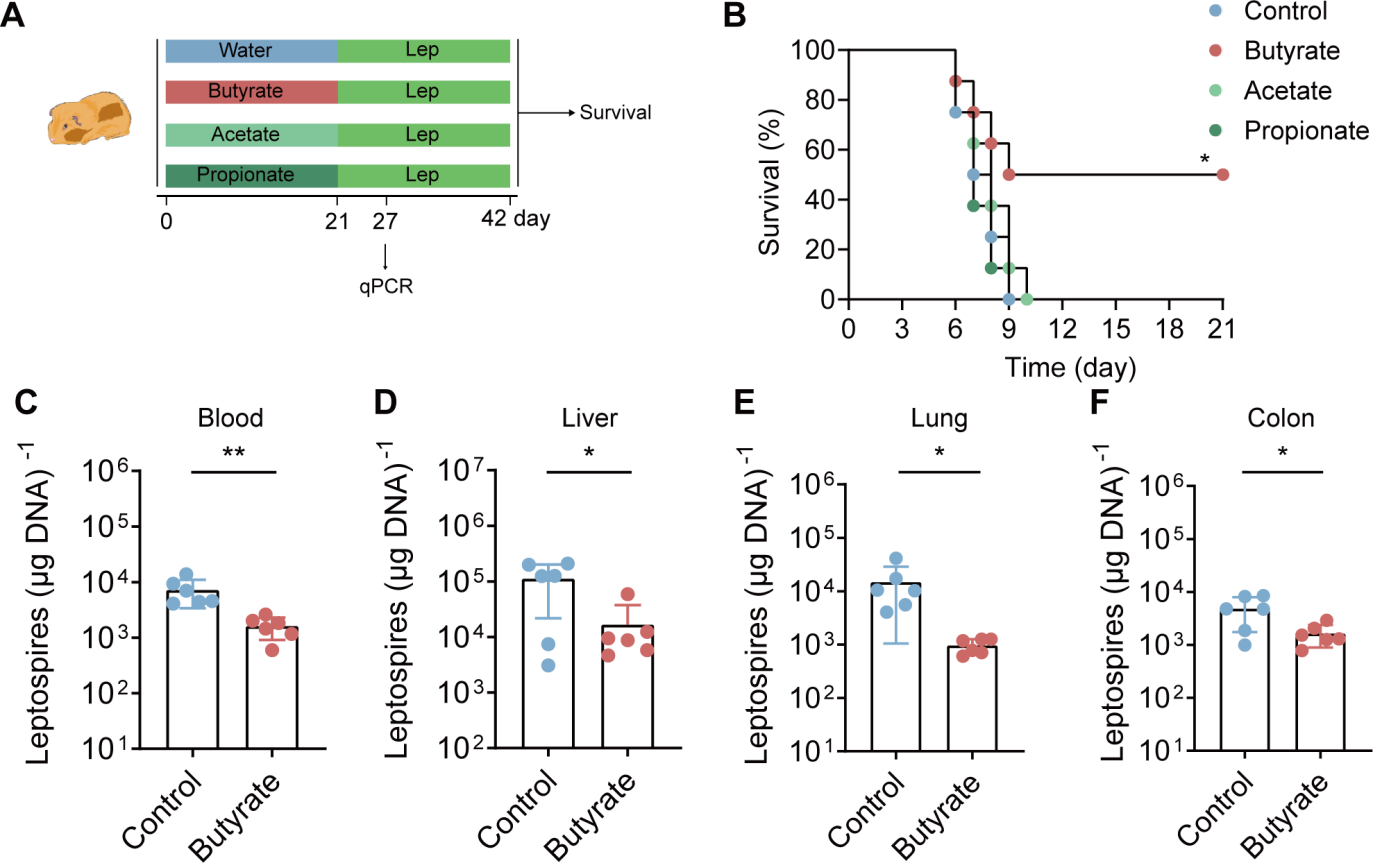
SCFAs butyrate improves the survival rate of leptospirosis in hamsters. (**A**) Flow diagram of the experiment. Acetate (150 mM), propionate (150 mM), and butyrate (150 mM) were added to the drinking water of hamsters, and then the hamsters were infected with 1 × 10^6^ leptospires for 21 days (*n* = 6). The blood, livers, lungs, and colons of the butyrate group and the control group were collected on the sixth day of infection. (**B**) Survival curves of the acetate group (*n* = 8), propionate group (*n* = 8), butyrate group (*n* = 8), and control group (*n* = 8) after infection with 1 × 10^6^ leptospires. (**C**) The leptospiral burden in the blood (**C**), livers (**D**), lungs (**E**), and colons (**F**) was detected by qPCR (*n* = 6). Each experiment was repeated three times. The data are shown as the mean ± SEM. Statistical significance was determined using the Wilcoxon rank-sum test. *P < 0.05. **P < 0.01.

To confirm the role of SCFAs in leptospirosis and which SCFAs have the greatest effect on *Leptospira* infection, we administered acetate, propionate, and butyrate in water for 3 weeks (Fig. 2A). The results showed that butyrate significantly improved the survival rate of hamsters after *Leptospira* infection (Fig. 2B). In addition, compared with the control treatment, butyrate treatment reduced the bacterial load in the blood, livers, lungs, and colons (Fig. 2C through F). However, acetate and propionate treatment had no effect on the survival rate of the hamsters (Fig. 2B). Previous studies have reported that butyrate can directly regulate the pathogenicity of *Salmonella* (25). The *Leptospira* was cultured in EMJH medium with (or without) butyrate for 5 days. Then, infected hamsters were tested for hamster survival to determine the effect of butyrate on the pathogenicity of *Leptospira. Leptospira* RNA was extracted to detect the effect of butyrate on the expression of *Leptospira* virulence gene. The results showed that butyrate treatment did not change the expression of *Leptospira* virulence genes (Fig. S1A and B). In addition, there was no significant difference in the survival rate of hamsters infected with *Leptospira* (with or without butyrate) (Fig. S1C). The result suggests that butyrate does not affect the virulence of *Leptospira*. In conclusion, gut microbiota-derived butyrate protects the host against acute leptospirosis.

### Butyrate enhanced the antibacterial activity of macrophages by promoting the production of ROS after infection

**Fig 3:**
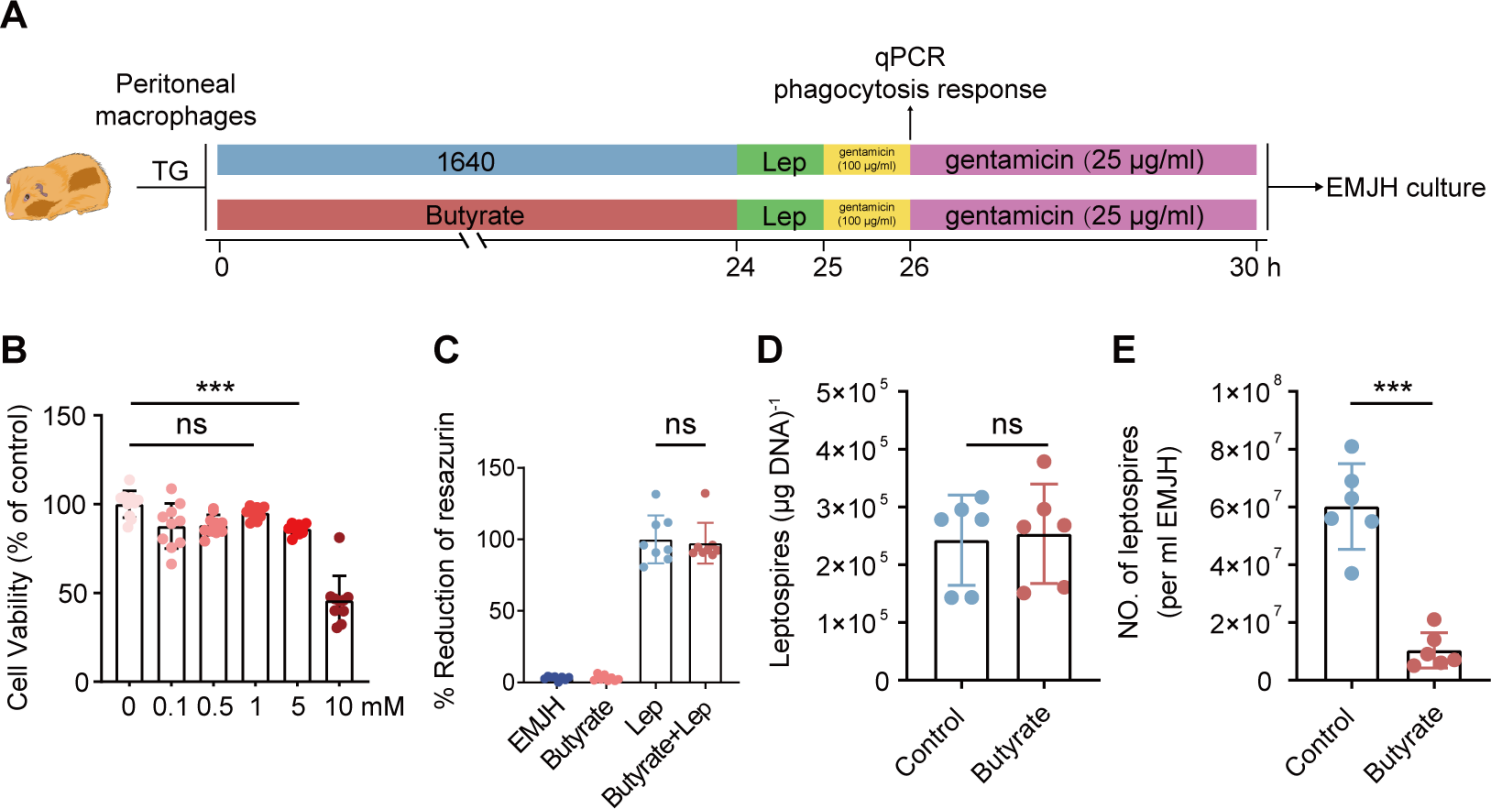
Butyrate treatment enhanced the antimicrobial capacity of hamster macrophages (**A**). Flow diagram of the experiment. TG macrophages were isolated from hamsters. After being cultured in a medium containing (or without) butyrate (1 mM) for 24 h, a total of 2 × 10^6^ cells were infected with leptospires at a multiplicity of infection (MOI) of 100. (**B**) Effect of butyrate on the proliferation of macrophages. (**C**) The percentage of resazurin reduction of butyrate incubated with leptospires (*n* = 6). (**D**) The phagocytic response of control group macrophages and the butyrate-treated group macrophages was analyzed by qPCR after 2 h of infection. (**E**) Gentamycin protection assay of control group macrophages and butyrate-treated group macrophages (*n* = 6). Each experiment was repeated three times. The data are shown as the mean ± SEM. Statistical significance was determined using the Wilcoxon rank-sum test. ***P < 0.001. ns not significant.

Previous studies have reported that macrophages play an important role in preventing leptospirosis (26, 27). Our recent study also proved that the gut microbiota could regulate anti-leptospiral activity by macrophages in mice (8). Thus, we wondered whether butyrate could enhance the anti-leptospiral activity of macrophages. The peritoneal cavity has been used as a typical source of primary cultured cells for examining multiphasic aspects of macrophage biology (14). Therefore, thioglycolate-induced (TG) peritoneal macrophages from hamsters were utilized to detect the effect of butyrate on the antibacterial activity of macrophages (Fig. 3A). A substantial amount of butyrate can be taken up by epithelial cells and 1 mM butyrate is often used to determine the amount of butyrate that might reach macrophages in the lamina propria from the colonic lumen (28). A series of concentrations ranging from 0 to 10 mM were used to test the effect of butyrate on macrophages. Treatment with butyrate at a concentration greater than 1 mM for 24 h significantly reduced cell viability compared with that of the controls (Fig. 3B). Butyrate also did not inhibit the growth of *Leptospira in vitro* (Fig. 3C). Thus, a butyrate concentration of 1 mM was used for the subsequent experiments.

The results showed that the phagocytosis (Fig. 3D) of macrophages in the butyrate group was comparable to those in the control group. Interestingly, the intracellular bactericidal ability of macrophages in the butyrate group was significantly greater than that in the control group (Fig. 3E). These results indicated that butyrate enhanced the intracellular bactericidal ability of macrophages to eliminate *Leptospira* infection in macrophages.

**Fig 4:**
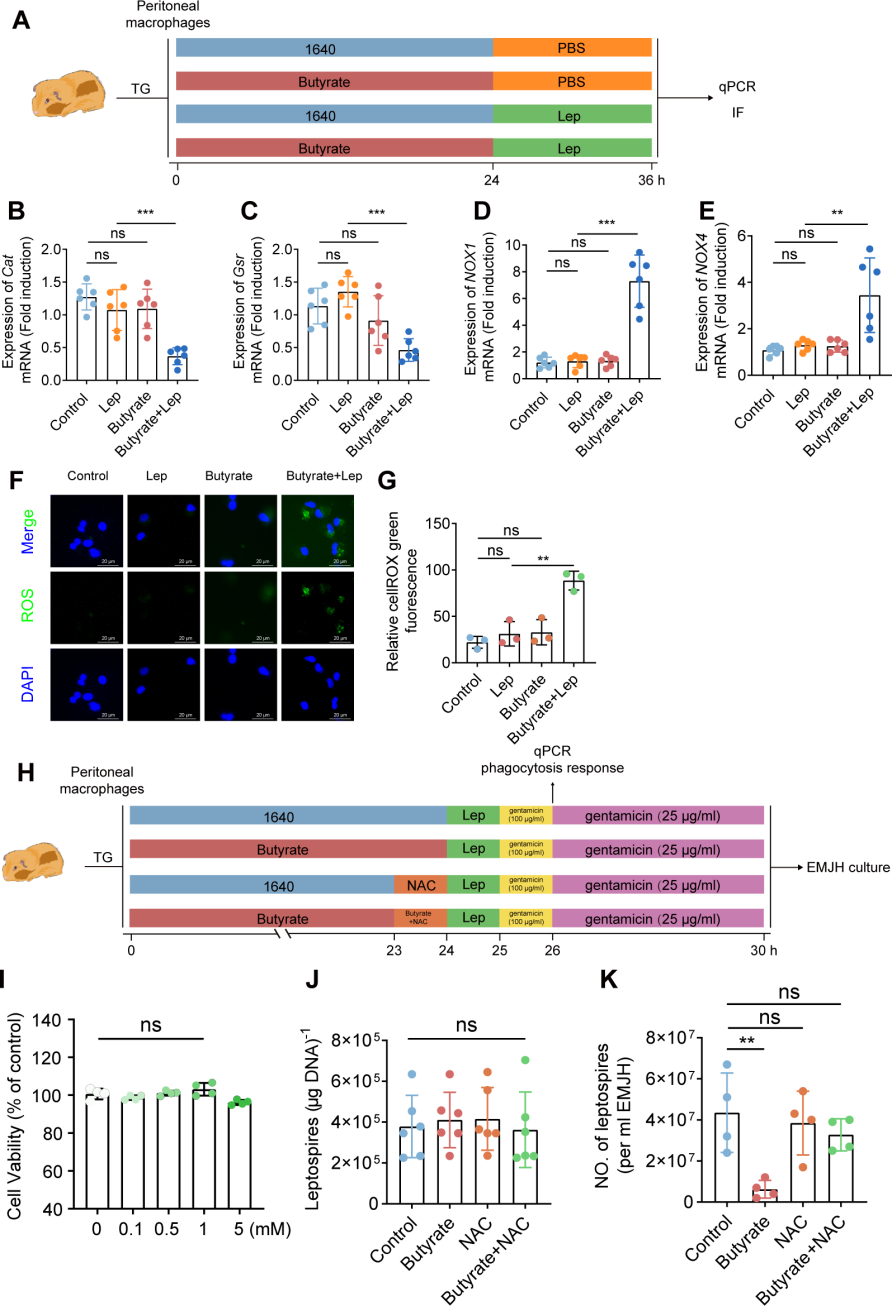
ROS expression and bactericidal function of butyrate-treated macrophages. (**A**) Flow diagram of the experiment. The TG macrophages were isolated from hamsters. Macrophages were infected with leptospires at a multiplicity of infection (MOI) of 100 with (or without) butyrate (1 mM) treatment for 24 h. (**B**) Macrophages were infected with leptospires for 12 h, and the gene expression levels of *Cat* (**B**), *Gsr* (**C**), *NOX1* (**D**), and *NOX4* (**E**) were examined *via* qPCR (*n* = 6). (**F**) Macrophages were infected with leptospires for 12 h, and ROS levels were examined by ROS Oxidative Stress in control hamsters and the butyrate-treated hamsters. (**G**) Quantification of ROS fluorescence intensity. (**H**) Flow diagram of the experiment. Thioglycolic acid-induced macrophages were isolated from hamsters. After the cells were cultured in a medium supplemented with (or without) butyrate (1 mM) for 23 h, then added (without NAC) NAC in the medium for 1 h, a total of 2 × 10^6^ cells were infected with leptospires at an MOI of 100. The protective effect of butyrate on the bactericidal function of macrophages was detected *via* gentamicin protection experiments. (**I**) Effect of NAC on the proliferation of macrophages. (**J**) The phagocytic response of macrophages in control hamsters and the butyrate-treated hamsters was analyzed by qPCR after 2 h of infection (*n* = 6). (**K**) Gentamycin protection assay of macrophages from control hamsters and the butyrate-treated hamsters (*n* = 4). Each experiment was repeated three times. The data are shown as the mean ± SEM. Statistical significance was determined using the Wilcoxon rank-sum test. **P < 0.01. ***P < 0.001. ns not significant.

We next sought to examine the underlying mechanisms whereby the intracellular anti-leptospiral response to *Leptospira* infection is enhanced by butyrate. Research shows that the generation of ROS by macrophages is an essential bactericidal mechanism during *Leptospira* infection (29). In addition, under bacterial stimulation, the bactericidal activity of macrophages increases with increasing butyrate concentration *via* the production of ROS, which favors bactericidal activity at the implant-associated infection stage (30). Therefore, we proposed that butyrate enhanced the intracellular anti-leptospiral activity of macrophages through ROS production. To validate this hypothesis, we first examined the expression of genes related to ROS production by qPCR (Fig. 4A). The results showed that butyrate inhibited the expression of *Cat* and *Gsr* in macrophages (Fig. 4B and C), but increased the expression of *NOX1* and *NOX4* after *Leptospira* infection (Fig. 4D and E). It is worth noting that by itself butyrate (without *Leptospira* infection) had no significant effects on the expression of *Cat*, *Gsr*, *NOX1*, and *NOX4*. Macrophage ROS staining also confirmed that butyrate treatment promoted the production of ROS after *Leptospira* infection (Fig. 4F and G). These results suggested that ROS may be the key to butyrate to enhance the antileptospiral ability of macrophages. In addition, acetate and propionate did not affect the ROS levels of macrophages after infection with *Leptospira* (Fig. S2A and B). To further determine the role of ROS in the protective effect of butyrate against *Leptospira* infection, we stimulated macrophages with ROS inhibitor N-Acetylcysteine (NAC) and butyrate (Fig. 4H). The results showed that NAC had no effect on the macrophages’ activity (Fig. 4I) while NAC diminished the enhanced anti-leptospiral activity of macrophages triggered by butyrate (Fig. 4J and K). These results indicated that butyrate enhanced the anti-leptospiral ability of macrophages by promoting the production of ROS.

### Butyrate promoted the production of ROS in macrophages through its HDAC3 inhibitory function mediated by MCT

**Fig 5:**
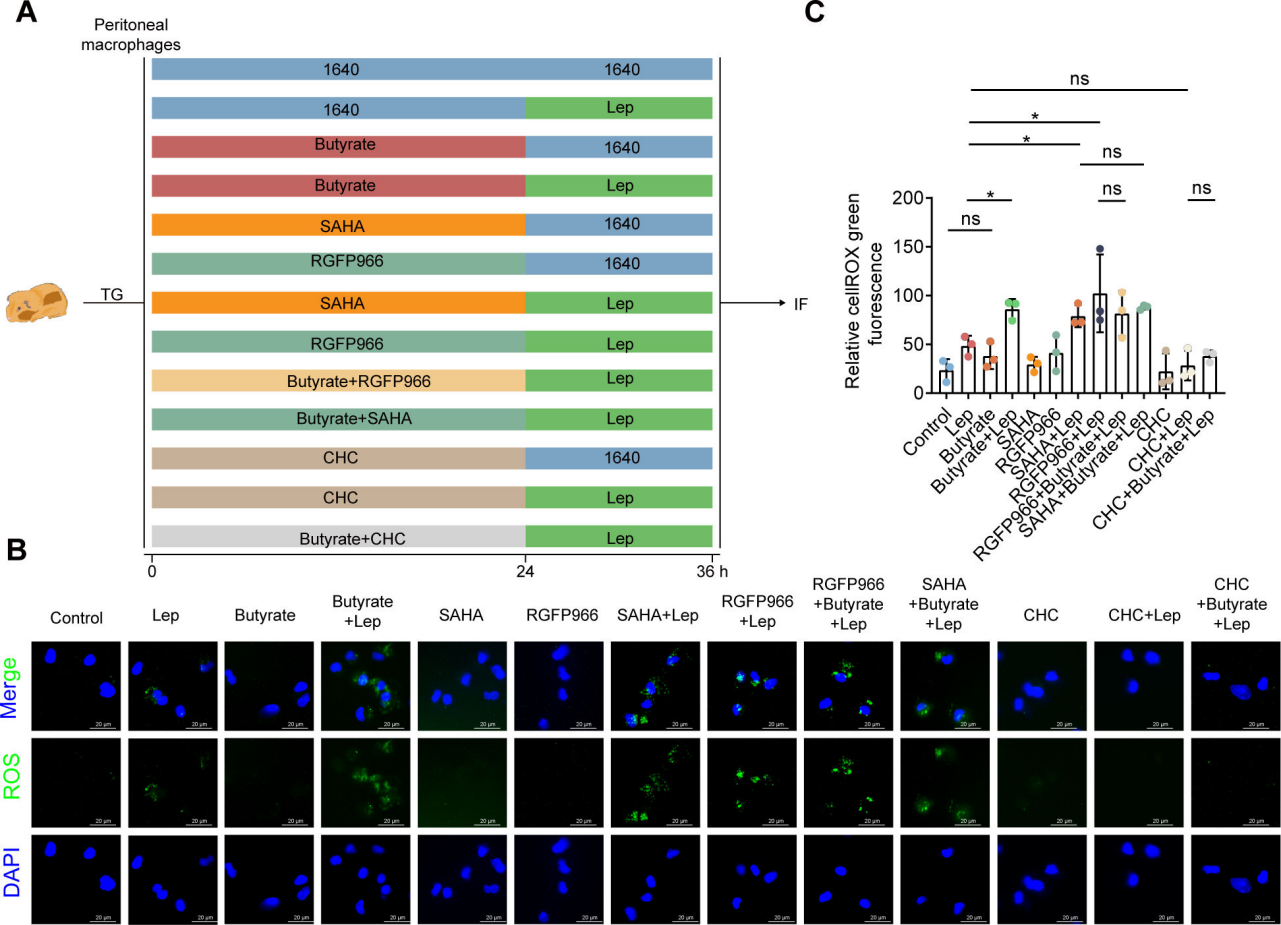
Butyrate increased macrophage ROS levels through HDACi activity. (**A**) Flow diagram of the experiment. ROS levels in the macrophages in the butyrate group, RGFP966 group, SAHA group, and CHC (α-cyano-4-hydroxycinnamate) group after infection with 10^8^ leptospires for 12 h. (**B**) The ROS levels in macrophages treatment with butyrate, RGFP966, SAHA, and CHC with (or without) *Leptospira* infection. (**C**) Quantization of ROS fluorescence intensity. Each experiment was repeated three times. The data are shown as the mean ± SEM. Statistical significance was determined using the Wilcoxon rank-sum test. *P < 0.05. ns not significant.

The next question we asked was how butyrate induced ROS production after *Leptospira* infection. Previous studies have shown that epigenetic modifications are correlated with the overproduction of ROS. HDAC inhibitors are powerful epigenetic regulators that have pleiotropic effects at the cellular and systemic levels (31, 32). Butyrate has a well-known role as an HDAC inhibitor (HDACi), and we reasoned that it was transported by MCT to function as an HDACi to regulate the production of ROS. To test this hypothesis, we used α-cyano-4-hydroxycinnamate (CHC), an inhibitor of MCT (Fig. 5A). The results showed that CHC treatment almost completely eliminated the increase in ROS production induced by butyrate after *Leptospira* infection (Fig. 5B and C). This finding suggested that butyrate acted intracellularly. We next tested the role of the HDAC inhibitory function of butyrate in enhancing ROS function. HDACs remove acetyl groups on specific lysine residues from histones and non-histone proteins regulating gene expression by modulating chromatin structure (13). To study the functional effect of HDAC inhibition, we used the pan-HDAC inhibitor SAHA (Fig. 5A). The results showed that SAHA could also increase the production of ROS in macrophages after *Leptospira* infection (Fig. 5B and C). In addition, there were no additional effects of butyrate on SAHA-treated macrophages’ ROS levels after *Leptospira* infection. These findings suggested that butyrate might regulate ROS production in macrophages after infection with *Leptospira* by inhibiting the activity of HDACs. We next investigated the specificity of butyrate-mediated HDAC inhibition in macrophages. Compared with those in the control group, the ROS production in the group treated with RGFP966 (an HDAC3-specific inhibitor) increased after *Leptospira* infection (Fig. 5B and C). Although ROS production was promoted by butyrate or RGFP966 treatment after *Leptospira* infection, there were no additional effects of butyrate on RGFP966-treated macrophage ROS level after *Leptospira* infection. These data demonstrated that the butyrate-mediated increase in ROS production of macrophages was dependent mainly on HDAC3i.

### Butyrate protected hamsters against *Leptospira* infection by promoting ROS production

**Fig 6:**
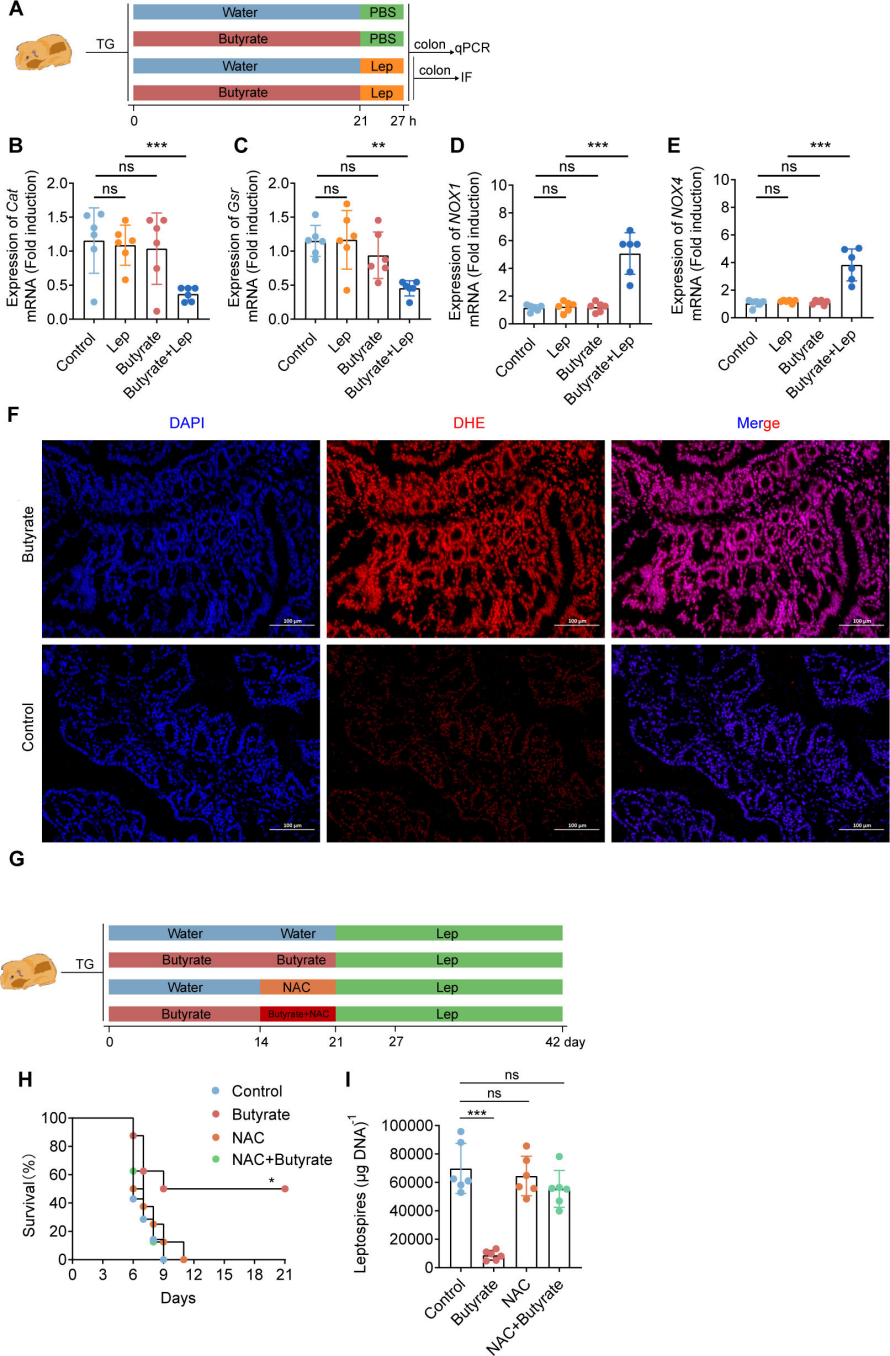
Butyrate protected hamsters against *Leptospira* infection by promoting ROS production. (**A**) Flow diagram of the experiment. Butyrate (150 mM) was added to drinking water for 21 days, after which the female hamsters were infected with leptospires. The colons were collected 6 days later. (**B**) qPCR was used to detect the expression of the *Cat* (**B**), *Gsr* (**C**), *NOX1* (**D**), and *NOX4* (**E**) genes in the colon (*n* = 6). (**F**) Colonic tissue ROS staining. (**G**) After drinking butyrate for 14 days, NAC was added to water with (or without) butyrate for another 7 days, then each hamster was infected with 10^6^ leptospires. (**H**) Survival curve of hamsters with infection with leptospires in the control group, butyrate-treated group, NAC group, and butyrate + NAC group (*n* = 8). (**I**) qPCR was used to detect the expression of the leptospiral load in the colons of in the control group, butyrate-treated group, NAC group, and butyrate + NAC group (*n* = 8). The data are shown as the mean ± SEM. Statistical significance was determined using the Wilcoxon rank-sum test. *P < 0.05. **P < 0.01. ***P < 0.001. ns not significant.

Based on the above results, we wondered whether the protective effect of butyrate against *Leptospira* infection *in vivo* was mediated by ROS production. To test this hypothesis, we added 150 mM butyrate to the drinking water of hamsters and infected them with 10^6^ leptospires at 21 days (Fig. 6A). The results showed that *Cat* and *Gsr* were suppressed and *NOX1* and *NOX4* were upregulated in the butyrate-treated hamsters after *Leptospira* infection (Fig. 6B through E). The results of colonic tissue staining showed that ROS level in the butyrate treatment group was significantly greater than that in the control group (Fig. 6F). The above experimental results indicated that the upregulation of ROS was the key factor through which butyrate enhances the bactericidal ability of macrophages, which may also explain why butyrate protects the host by reducing the bacterial load and improving the survival rate of sensitive hosts. To test this hypothesis, we added the ROS inhibitor NAC to the drinking water of hamsters (Fig. 6G). The results showed that there was no significant difference in the survival rate and leptospiral load between the NAC-inhibited group and the control group. The survival rate of the butyrate treatment group was greater than that of the NAC group, NAC + butyrate treatment group, or control group hamster (Fig. 6H). Besides, the leptospiral load of the colon was lower in the butyrate treatment group hamster than in the NAC treatment group, NAC + butyrate treatment group, or control group hamster (Fig. 6I). Taken together, our data reveal the importance of the MCT-HDAC3i-ROS axis in mediating the protective effect of butyrate against *Leptospira* infection.

## DISCUSSION

To date, the major treatment method for leptospirosis is antibiotics but late leptospirosis leads to multiple organ dysfunction and Jarisch-Herxheimer reaction is also a risk factor in antibiotic treatment. Thus, it is imperative to explore new and effective agents for leptospirosis treatment and prevention. Our previous study demonstrated that the gut microbiota helps mice resist *Leptospira* infection (8). However, the interaction mechanism between the gut microbiota and host immunity has not been thoroughly studied. In this study, we found that gut microbial-derived SCFAs had a protective effect against hamster leptospirosis. The SCFA butyrate enhances the bactericidal activity of macrophages by promoting the production of ROS.

The gut microbiota can maintain the dynamic immune balance, while a loss of healthy microbiota (biological imbalance) may lead to an increase in susceptibility to infectious diseases (33). This finding is consistent with our finding that the depletion of the gut microbiota leads to a significantly accelerated leptospirosis in hamsters. The intestinal SCFAs are mainly acetate, propionate, and butyrate (34), which are produced by anaerobic fermentation in the intestinal tract. SCFAs produced in the intestinal cavity are easily absorbed by colon cells and used as energy (35). In addition, butyrate is involved in regulating cell proliferation and differentiation, hormone secretion, and immune/inflammatory activation (36–38). SCFAs are involved in the regulation of a variety of immune cell functions and inflammatory responses. A previous study reported that SCFAs are involved in the regulation of leukocyte recruitment and chemokine expression (35, 39). SCFAs also affect the production of cytokines by immune cells, but the mechanisms differ among different cells (35, 40). Interestingly, butyrate enhanced the antibacterial activity of macrophages by inhibiting histone deacetylase 3 (13), which attracted our attention because macrophages play an important antibacterial role in *Leptospira* infection. Therefore, we analyzed SCFAs in the feces of hamsters in the Abx group and the control group. GC-MS analysis of the feces of Abx hamsters revealed that the levels of several main SCFAs, such as acetate, propionate, and butyrate in the feces, were significantly lower than those in the healthy hamsters. This finding suggested that SCFAs from the healthy gut microbiota of hamsters may play a role in resistance to leptospirosis. Previous studies showed that butyrate protected against severe influenza infection by boosting anti-viral immunity through the gut-lung axis (41). In addition, butyrate exerted a preventative effect against MRSA-induced pneumonia by promoting macrophage polarization toward an anti-inflammatory M2 phenotype through the gut-lung axis (41). A recent study also reported that butyrate alleviated *Staphylococcus aureus*-induced mastitis by activating the antimicrobial program through the gut-mammary gland axis (42). These studies suggest that butyrate could play a role in regulating host immunity against systemic infection (43).

To further determine the role of butyrate in leptospirosis in hamsters, we pretreated hamsters with butyrate for 3 weeks and infected them by *Leptospira*. The results showed that butyrate significantly increased the survival rate of hamsters and reduced the leptospiral burden in the blood, livers, lungs, and colons. These results suggest that butyrate can enhance hamsters against leptospirosis. Intestinal is a common but neglected Target organ in severe leptospirosis (4, 44). We found that butyrate significantly inhibited the gene expression of *Gsr* and *Cat* in the colons, but upregulated the expression of the *NOX1* and *NOX4* genes. The former is the core enzyme of cellular antioxidant defense (45, 46), while the latter is NADPH oxidase, which can induce neutrophils and macrophages to produce ROS. We found that the level of ROS in the colon tissue of the butyrate-treated group was greater than that in the control group after *Leptospira* infection, which was consistent with the gene expression results. Interestingly, ROS are important means by which macrophages kill pathogens, suggesting that butyrate may enhance host immune killing by promoting the expression of ROS in macrophages. Our study revealed that butyrate protected hamsters from *Leptospira* infection.

In infectious diseases, neutrophils and macrophages internalize, kill, and digest invasive pathogens by producing reactive oxygen species and releasing granzymes (47, 48). In patients with *Leptospira* infection, macrophages are the main immune cells responsible for clearing leptospires (27). Leptospires have been shown to survive and reproduce in the macrophages of sensitive hosts, while leptospires can be killed in the macrophages of tolerant animals (49). The differential response of macrophages to leptospirosis may be one of the reasons for the different severities of leptospirosis in heterogeneous animals. In this study, we found that butyrate did not increase the efficiency of macrophage phagocytosis of leptospires but did increase the bactericidal function of macrophages. The enhancement of the bactericidal function of macrophages may be the main reason why butyrate enhances the resistance of hamsters to *Leptospira* infection. During phagocytosis of pathogens, macrophages engulf them into phagosomes, fuse phagosomes with lysosomes to form phagosomes, and finally kill and degrade pathogens by phagocytosing acidic environments, ROS, nitric oxide (NO), and hydrolases. We found that the *Cat* and *Gsr* genes were inhibited in butyrate-treated macrophages, but the *NOX1* and *NOX4* genes were induced. Similar to the gene expression results, the ROS level in butyrate-treated macrophages after phagocytosis of leptospires was greater than that in the control group. The antioxidant NAC (which inhibits ROS production in cells) was used to stimulate macrophages simultaneously with butyrate. The results showed that NAC reduced the bactericidal function of macrophages enhanced by butyrate, suggesting that butyrate enhanced the bactericidal function of macrophages by increasing ROS production. Interestingly, we found that acetate and propionate did not affect ROS production in macrophages after infection with *Leptospira*. This may also explain why acetate and propionate do not protect the host against acute leptospirosis like butyrate.

Our results showed that butyrate enhanced the bactericidal activity of macrophages by promoting the production of ROS. Butyrate affects the activity of HDACs. HDACs are a class of histone-modifying enzymes that regulate gene transcription and have the potential to affect biological processes. We used monocarboxylic acid transporter inhibitor CHC and butyrate to stimulate macrophages. The results showed that CHC inhibited the ability of butyrate to regulate the expression of ROS in macrophages. This finding shows that butyrate does not rely on membrane receptors. We found that the HDAC3 inhibitor RGFP966 could increase the expression of ROS in macrophages as well as butyrate. Moreover, the simultaneous stimulation with RGFP966 and butyrate did not further increase the expression of ROS, indicating that butyrate regulates the expression of ROS in macrophages by inhibiting HDAC3.

**Fig 7:**
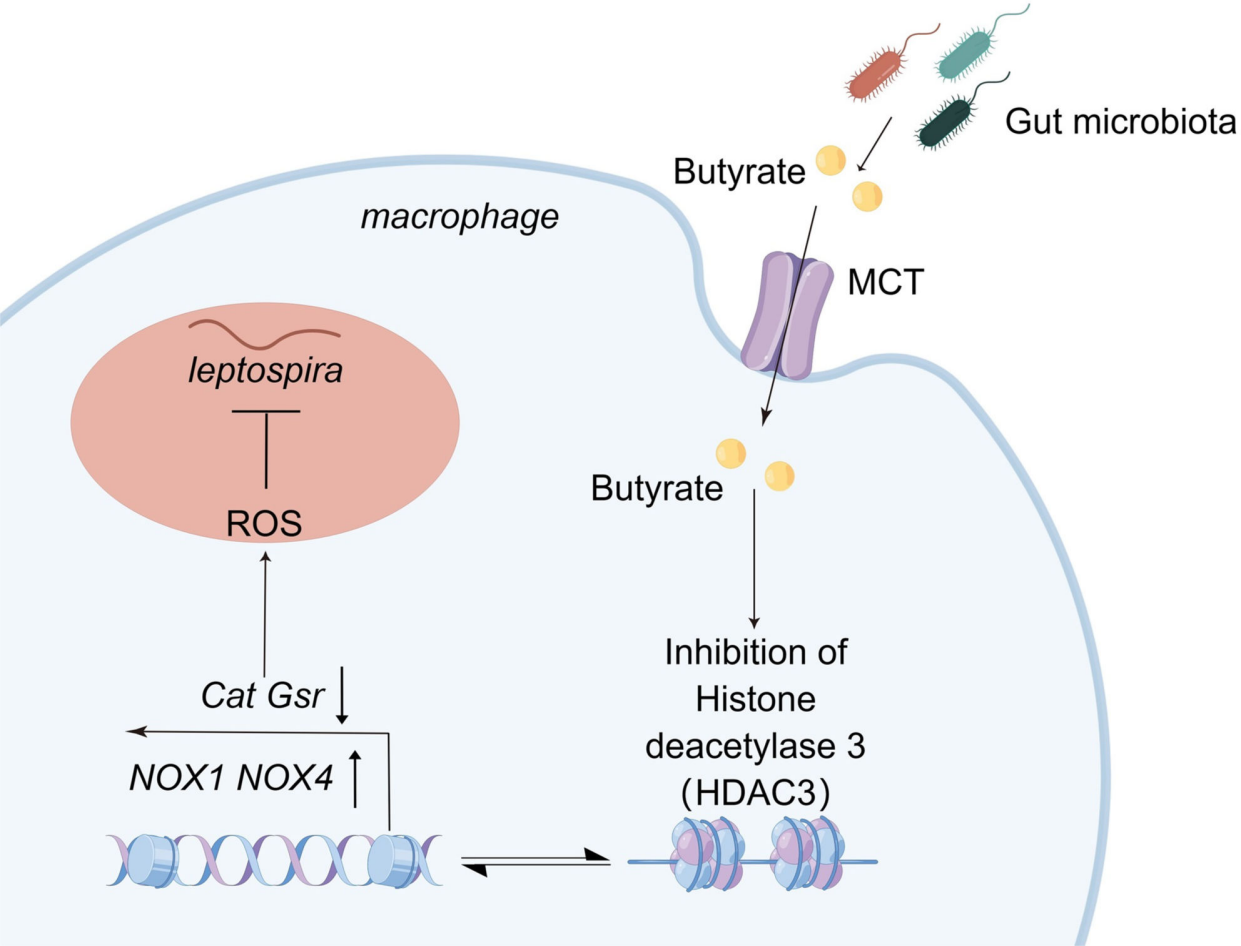
The microbiota metabolite butyrate protected against acute leptospirosis by macrophage ROS production *via* HDAC3 inhibition in hamsters. Butyrate from the gut microbiota enhances macrophage antibacterial activity. The butyrate produced by the gut microbiota is transported into macrophages by MCT. Intracellular butyrate increases the expression of ROS in macrophages by regulating gene expression of *Cat*, *Gsr*, *NOX1*, and *NOX4*, and this process is mediated by the HDACi of butyrate.

In summary, the prevention and treatment of leptospirosis are limited. Conventional antibiotic treatment is only aimed at early leptospirosis (50), and the use of antibiotics can cause disorders of the gut microbiota, leading to opportunistic infections caused by *Clostridium difficult*, *Clostridium perfringens*, and *Staphylococcus aureus*, resulting in mucosal immune disorders and diarrhea diseases (51, 52). Our study showed that the gut microbiota is an endogenous barrier against leptospirosis. Butyrate from the gut microbiota can enhance the bactericidal function of macrophages by inhibiting HDAC3 to regulate the expression production of ROS (Fig. 7). Our work not only interprets the microbial metabolite signals involved in transkingdom interactions between the host and gut microbiota but also provides a new and effective prevention strategy for leptospirosis.

## SUPPLEMENTAL MATERIAL

Supplemental figures mbio.01906-24-s0001.docx Fig. S1 and S2.

ASM does not own the copyrights to Supplemental Material that may be linked to, or accessed through, an article. The authors have granted ASM a non-exclusive, world-wide license to publish the Supplemental Material files. Please contact the corresponding author directly for reuse.
