# Supplementary material for: Gut microbiota-derived butyrate improved acute leptospirosis in hamster via promoting macrophage ROS mediated by HDAC3 inhibition
Source: mBio. 2024 Sep 17;15(10):e01906-24. doi: 10.1128/mbio.01906-24 (PMC11481532; doi:10.1128/mbio.01906-24)
Supplement: Supplemental figures — Fig. S1 and S2. [file mbio.01906-24-s0001.docx]

**
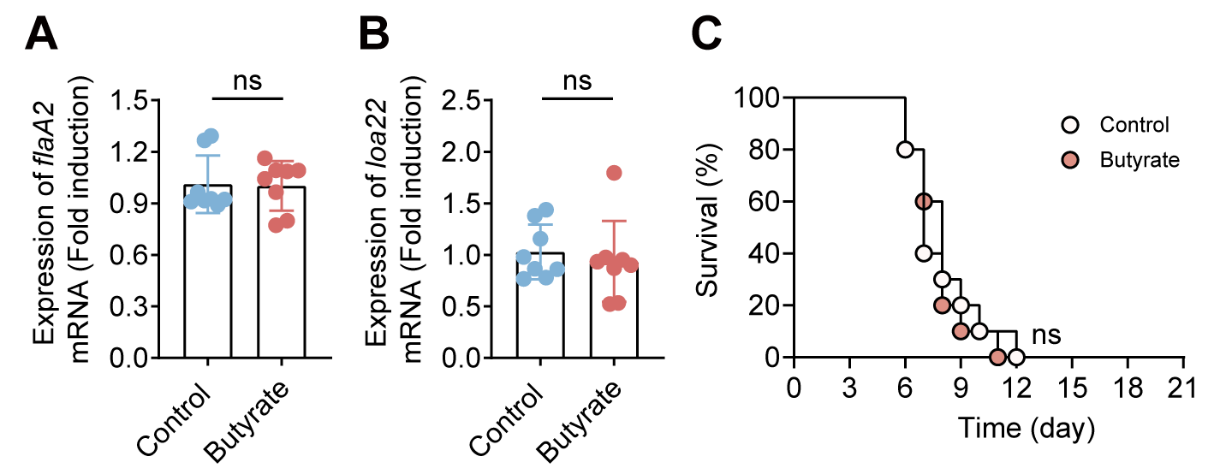
**

**Supplementary Fig. S1. Effect of butyrate on virulence of *Leptospira*. A**. The gene expression levels of *flaA2* (**A**) and *loa22* (**B**) in *Leptospira* (cultured in EMJH medium with or without butyrate) (n=8). **C.** Survival rate of hamsters (n=8) after infected with 10^6^ leptospires (cultured in EMJH medium with or without butyrate). The data are shown as the mean ± SEM. Statistical significance was determined using the Wilcoxon rank-sum test. ns not significant.


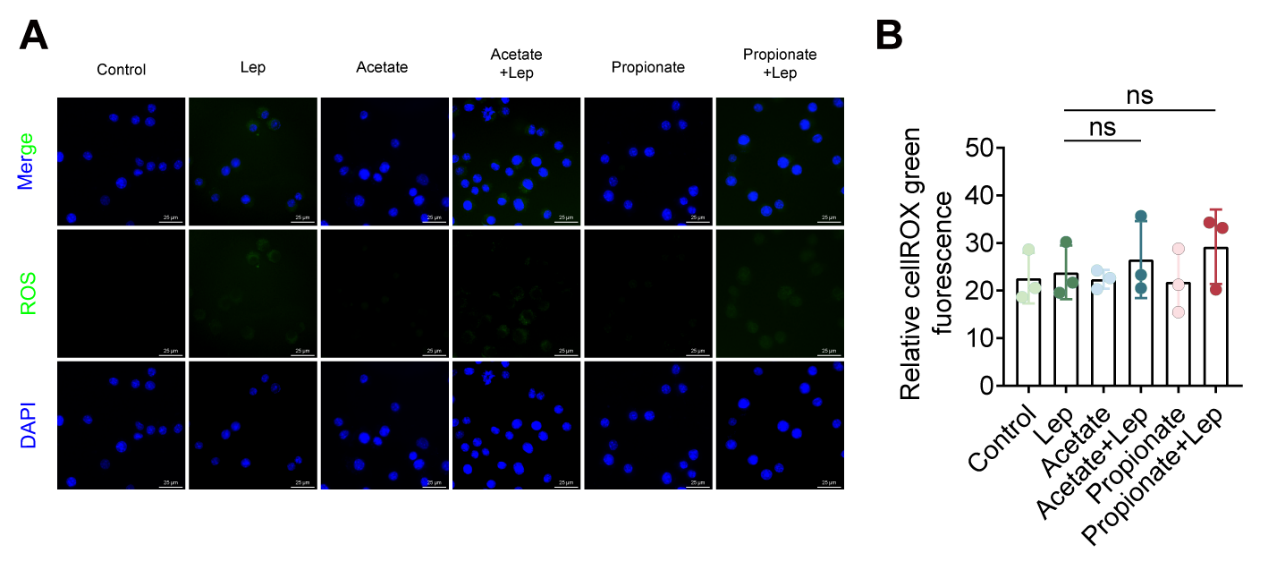


**Supplementary Fig. S2. Effects of acetate and propionate on ROS levels in macrophages. A.** The ROS levels in macrophages treatment with acetate and propionate with (or without) *Leptospira* infection. **B.** Quantization of ROS fluorescence intensity (n=3). The data are shown as the mean ± SEM. Statistical significance was determined using the Wilcoxon rank-sum test. ns not significant.
